# Treatment of Generalized Granuloma Annulare with Oral Griseofulvin

**DOI:** 10.1155/2022/2523710

**Published:** 2022-11-18

**Authors:** Chumsaeng Chumsaengsri, Jongjin Suwanthaweemeesuk

**Affiliations:** Institute of Dermatology, Bangkok, Thailand

## Abstract

**Background:**

Granuloma annulare (GA) is a benign skin disease that has four clinical variants including localized, generalized, perforating, and subcutaneous GA. The most common type is localized GA, followed by generalized GA. Generalized GA was defined as at least 10 widespread annular plagues and frequently on the trunk, face, neck, and extremities. The diagnosis was made by clinical and histopathology. Generalized GA was difficult to treat. *Case Presentation*. We presented a Thai woman with nonscaly annular papules and plaques on the trunk and all extremities. A skin biopsy revealed a lesion that was compatible with granuloma annulare. She was partially resolved with 2-month course of oral griseofluvin 500 mg daily. *Discussion*. The regression of GA response to oral griseofulvin is consistent with the inflammatory nature, which identified IFN-gamma upregulated in GA.

**Conclusion:**

Griseofulvin is safe with few side effects and cost effectiveness. Further studies are needed to better understand the immunology and pathogenesis of GA.

## 1. Introduction

Granuloma annulare (GA) is an inflammatory granulomatous skin disease affecting all ages but most frequently during the fifth decade of life and more common in women [1]. Although the pathogenesis of GA is unclear, it represents a benign cutaneous reaction with a variety of trigger factors. Infectious agents including both viral and bacterial triggers, several vaccinations, medications, bee string, octopus bite, and red pigmented tattoo were leading to the development of GA [2]. Drug-induced granuloma annulare are typically resolved when discontinuing the culprit drug, and drug lists including angiotensin-converting enzyme inhibitors, anti-TNF agents (infliximab, adalimumab, etanercept, and lenalidomide), diuretics (furosemide), gold, methotrexate, and immunization (hepatitis B and anti-tetanus vaccination) [3]. Hyperlipidemia and hypothyroidism remained significantly associated with GA while type II diabetes mellitus was not a significant association [4].

A recent publication from New York reported the upregulation of T-helper cell type 1 (tumor necrosis factor (TNF)-alpha, interleukin (IL)-1beta, IL-12/23p40), and T-helper cell type 2 (IL4 and IL31) pathways in GA lesional skins compared with normal skin of healthy adults [5]. Furthermore, the significant upregulation of the Janus kinase signal transducer and activator of transcription (JAK-STAT) pathway in GA lesional skin [5]. Another recent publication reported JAK-STAT dependent cytokines, interferon (IFN)-gamma, and oncosatin M are upregulated in GA lesions, as well as IL-21 and IL-15 [6]. GA is T-cell-dependent disorder and the immune mechanism could be driving this disease [5, 6].

Currently, there are no effective treatments for GA due to lack of pathogenesis. The main purpose of treatment is to relieve pruritus and improve quality of life. Generalized GA is recalcitrant to therapy [5]. Multiple therapeutic options for generalized GA including hydroxychloroquine [7], PUVA [8], dapsone [9], methotrexate [10], TNF-alpha inhibitor (adalimumab [11] and dupilumab [12]), apremilast [13], and JAK inhibitor (tofacitinib [14]) have been reported to be effective. Around 50% of patients spontaneously resolve within 2 years, and recurrence is also common [2].

## 2. Case Report

**Figure 1:**
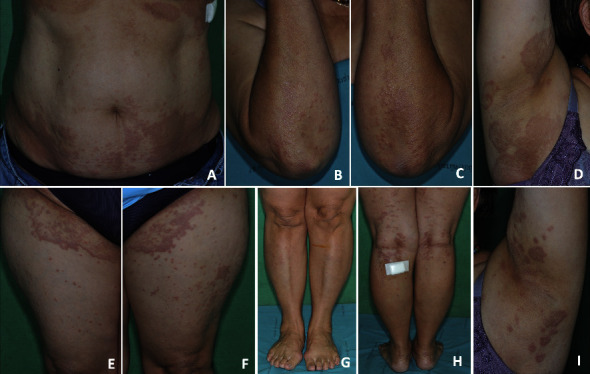
Clinical features: multiple well-defined nonscaly, erythematous annular papules, and plaques on the trunk, both axillar, both groin, and all extremities (a–i).

A 58-year-old Thai woman came to the outpatient clinic with pruritic, nonscaly erythematous annular papules and plaques on the trunk, both axillar, both groin, and all extremities for 3 months. At first, she had this clinical manifestation 2 years ago and treated with methotrexate and topical corticosteroids at the previous hospital, but the symptoms did not improve. She still had extremely pruritic, numerous nonscaly erythematous papules, and plaques on the same area so she went to another hospital. She had the same treatment with methotrexate, and her symptoms did not improve. No triggers were reported. She had well-controlled hypertension on losartan 50 mg per day. Dermatological examination revealed the same clinical manifestation as previously mentioned (Figures 1(a)–1(i)). Lymph nodes can not be palpated.

**Figure 2:**
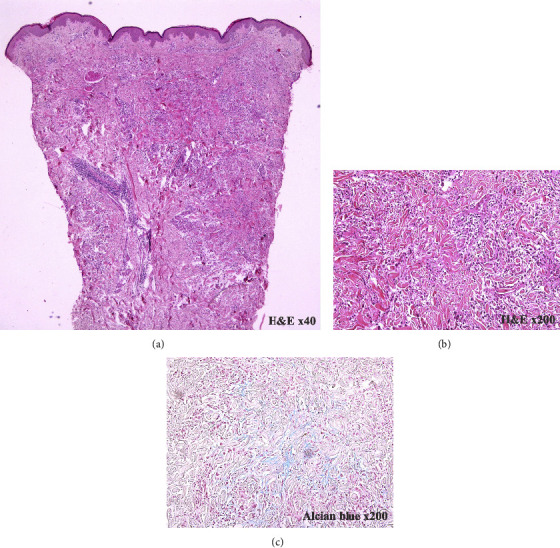
Histopathology showing palisading granuloma with mucin deposit (a and b). Alcian blue special stain is positive in the center of aggregate (c).

**Figure 3:**
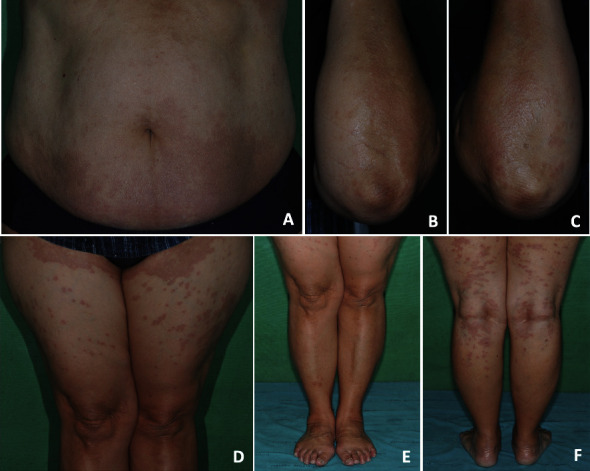
Clinical features of the improved patient: multiple well-defined nonscaly, erythematous annular papules, and plaques were faded in color (a–f).

A potassium hydroxide preparation was negative. The skin biopsy of the trunk and left leg showed a focal aggregate of histiocytes reminiscing palisading granuloma and degeneration of collagen in the center is noted. No inflammatory cell is present. Alcian blue special stain is positive in the center of the aggregate (Figures 2(a)–2(c)). Her blood test showed normal fasting blood glucose (FBS 97 mg/dL) and normal lipid profile (cholesterol level 200 mg/dL, triglyceride level 72 mg/dL). She was diagnosed with generalized granuloma annulare. She was prescribed oral griseofulvin 500 mg per day and follow-up one month later, the rash was still present but had faded in color, so we prescribed our patient topical low potency corticosteroids (0.05% betamethasone valerate) applied twice daily on the trunk and all extremities. At follow-up two months later, her symptoms were improved and the rash had faded in color (Figures 3(a)–3(f)).

## 3. Discussion

Generalized GA can persist for a decade despite therapy [11]. In our case, the clinicopathological data supported the provisional diagnosis of granuloma annulare. Blood sampling for screening metabolic disease was normal. No precipitated factor was reported.

Griseofulvin is known as an antifungal drug for many years and has antibacterial activity [15]. In the previous study, Griseofulvin has potent immunomodulatory properties which its feature was a microtubule antagonist that caused rapid, reversible, and repeated dissociation of mitotic spindle compared to colchicine [16]. Furthermore, griseofulvin inhibited IFN-gamma-induced HLA-DR expression on human keratinocytes is similar to colchicine [16]. This drug has been reported to be a benefit in lichen planus [17, 18] and pigmented purpuric dermatosis [19]. Griseofulvin is safe with few side effects and cost effectiveness. We prescribed our patient oral griseofulvin 500 mg per day and followed up two months later, her symptoms were partially improved. The regression of GA response to oral griseofulvin is consistent with the recent study by Wang et al. [6], which performed single cell RNA sequencing to identified IFN-gamma target genes and has been upregulated by macrophages in GA.

To the best of our knowledge, this is the first case report of generalized GA being successfully treated with oral griseofulvin. Further studies are needed to understand the pathogenesis of GA, approach the new target therapy, and need to be done to corroborate our preliminary findings.
